# Association between yogurt consumption and the risk of Metabolic Syndrome over 6 years in the SUN study

**DOI:** 10.1186/s12889-015-1518-7

**Published:** 2015-02-21

**Authors:** Carmen Sayón-Orea, Maira Bes-Rastrollo, Amelia Martí, Adriano M Pimenta, Nerea Martín-Calvo, Miguel A Martínez-González

**Affiliations:** Department of Preventive Medicine and Public Health, University of Navarra, Pamplona, Spain; CIBERobn Physiopathology of Obesity and Nutrition, Instituto de Salud Carlos III (ISCIII), Madrid, Spain; Department of Food Sciences and Physiology, School of Pharmacy, University of Navarra, Pamplona, Spain; Department of Maternal and Child Nursing and Public Health, Universidade Federal de Minas Gerais, Belo Horizonte, Brazil

**Keywords:** Metabolic syndrome, Yogurt, Fruit, Cohort studies

## Abstract

**Background:**

The role of yogurt consumption in the development of metabolic syndrome (MetS) is not fully understood and the available epidemiologic evidence is scarce. The aim of our study was to assess the association between total, whole-fat, or low-fat yogurt consumption and the risk of developing MetS.

**Methods:**

Yogurt consumption was assessed at baseline through a 136-item validated FFQ. MetS was defined following the harmonized definition for MetS according to the AHA and the IDF criteria. Logistic regression models were used.

**Results:**

During the first 6-y of follow-up of the SUN cohort, 306 incident cases of MetS were identified. Frequent consumption [≥875 g/week (≥7 servings/week) versus ≤ 250 g/week (2 servings/week)] of total, whole-fat and low-fat yogurt consumption showed non-significant inverse associations with MetS [OR = 0.84 (95% CI: 0.60-1.18); 0.98 (95% CI: 0.68-1.41); and 0.63 (95% CI: 0.39-1.02) respectively]. Only one component of the MetS, central adiposity, was inversely associated with total and whole-fat yogurt consumption [OR = 0.85 (95% CI: 0.74-0.98) and 0.85 (95% CI: 0.73-0.99) respectively]. In the joint assessment of exposure to total yogurt consumption and fruit consumption, those in the highest category of total yogurt consumption, and having a high fruit consumption (above the median ≥264.5 g/day) exhibited a significantly lower risk of developing MetS [OR = 0.61 (95% CI: 0.38-0.99)] compared with those in the lowest category of total yogurt consumption and had fruit consumption below the study median.

**Conclusion:**

No significant association between yogurt consumption and MetS was apparent. Only one component out of the 5 MetS criteria, central adiposity, was inversely associated with high yogurt consumption. The combination of high consumption of both yogurt and fruit was inversely associated with the development of MetS.

## Background

The term metabolic syndrome (MetS) refers to a combination of metabolic abnormalities that in the mid to long-term increase the risk of cardiovascular disease and type 2 diabetes [1]. The 5 abnormalities included in this syndrome are the following: low levels of high-density lipoprotein cholesterol (HDL-C) (<40 mg/dL and <50 mg/dL for men and women respectively, or treatment with condition-specific medication), hypertriglyceridemia (triglycerides ≥150 mg/dL or condition-specific medication), high blood pressure (systolic ≥130 and/or diastolic ≥85 mmHg or antihypertensive drug treatment in a patient with a history of hypertension), impaired glucose metabolism (fasting plasma glucose ≥100 mg/dL, specific medication, or previous diagnosis of type 2 diabetes), and central adiposity (in Europides, waist circumference ≥94 cm or ≥80 cm, for men and women respectively) [2]. The prevalence of MetS among adults in 2010 in Spain was 22.7% [3], and, according to Alberti et al. [4], the prevalence of this syndrome is dramatically growing world-wide, mainly due to the increasing prevalence of obesity and sedentary lifestyles. Therefore, nowadays MetS might be considered an important threat to public health.

Many risk and protective factors are associated with MetS. Some of the studied factors that might be involved in this syndrome are related to diet composition, including the consumption of dairy products. However, investigations in this field have yielded contradictory results [5-7]. In this context, there are some studies that have reported an association between MetS and dairy products as a whole [7-9], and some that described the independent effect of various dairy products (especially milk, yogurt and cheese) [6,10]. Concerning results on yogurt consumption, the conclusions obtained are all in the same direction suggesting a significant inverse association between yogurt consumption and the development of MetS [7,11]. However, other studies have reported non-significant associations [6,10]. Importantly, none of the studies that explored the association between MetS and yogurt have studied the potential different effects for whole-fat and low-fat yogurt consumption. In this context, Martinez-Gonzalez et al. recently studied the effects of whole fat and low fat yogurt consumption on weight gain and the risk of overweight/obesity, finding differences between the two types of yogurts [12].

A review of epidemiologic evidence relating dietary patterns and MetS concluded that no individual dietary component can be considered as fully responsible for the development of MetS, and it might be possible that the interaction between many components of the diet may be able to protect against the development of the MetS [13]. It has been suggested that different dietary patterns might be associated with the development of metabolic syndrome [14,15]. Regarding fruit and yogurt consumption, it has been found to have a protective effect against the risk of overweight/obesity [12]. Therefore, in order to expand the knowledge about nutritional determinants of the MetS, we used data from 8,063 university graduates enrolled in the Seguimiento Universidad de Navarra (SUN) project, with the aim to assess the association between total, whole-fat and low-fat yogurt consumption and the incidence of MetS, and secondly, to evaluate this same association taking into account the influence of other healthy dietary habits (such as fruit consumption and adherence to a Mediterranean dietary pattern), among young adults from a Mediterranean cohort.

## Methods

### Study population

The SUN project is a prospective, multipurpose, and dynamic cohort study conducted in Spain, which was originally designed to establish associations between diet and the occurrence of several diseases and chronic conditions including overweight and obesity [16]. Using biennial mailed questionnaires participants have been continually followed-up. Participants’ recruitment started in December 1999, and it is permanently open. All participants are university graduates, with ages ranging from 20 to 90 years.

For the present study, we included a subsample of the cohort with a minimum follow-up period of 6 years, therefore only those participants who were recruited before September 2006 could be included (n = 15,909). To avoid reverse causality bias, we excluded 4,084 participants who met at least one MetS criterion at baseline. As recommended in nutritional epidemiology, we also excluded 1,187 participants who reported values for total energy intake at baseline out of predefined limits (less than 800 kcal/d in men and 500 kcal/d in women or more than 4,000 kcal/d in men and 3,500 kcal/d in women). Further exclusions were 578 participants without any follow-up who were considered lost to follow-up (retention rate = 95%), and 1,997 participants who did not provide the relevant information about diagnostic criteria for the MetS at the 6th -year follow-up. After these exclusions a total of 8,063 participants were available for the final analysis. The retention rate of the study was above 95%. A comparison analysis between baseline characteristics of the participants who were included in the study, and those participants who did not have any information at Q_6, did not find meaningful differences between them (data not shown).

The study was approved by the Human Research Ethical Committee of the University of Navarra. Voluntary completion of the first self-administrated questionnaire was considered to imply informed consent.

### Dietary assessment

A semi-quantitative food frequency questionnaire (FFQ) was included in the baseline questionnaire. The FFQ was repeatedly validated in Spain [17-19] and includes 136 food items. Nutrient composition of specified portion sizes was multiplied by the frequency of consumption in order to calculate each nutrient score. Frequencies of consumption were measured in 9 categories (ranging from never/almost never, to >6 servings/day) for each food item, in order to assess habitual dietary intakes over the previous year. We defined a Mediterranean dietary pattern “a priori” using the score proposed by Trichopoulou et al. [20]. The traditional Mediterranean dietary pattern is characterized by abundant consumption of olive oil (the major source of fat), plant foods (vegetables, fruits, cereals, and nuts), fresh fruit as daily dessert, low-to moderate intake of dairy products (cheese and yogurt), fish and poultry, low intake of red meat, and regular moderate intake of wine generally consumed during meals. For the present study the Mediterranean dietary pattern was appraised combining 8 out of 9 of items of the original score (fruits and nuts; vegetables; fish; legumes; cereals; meat and meat products; alcohol; and the ratio monounsaturated fatty acids/saturated fatty acids). Since yogurt consumption is our exposure of interest, we excluded consumption of dairy products from the score. Therefore, the score ranged from 0 to 8 points. Food composition tables valid for Spain were used to update the nutrient databank [21].

### Yogurt consumption assessment

Frequency of whole-fat and low-fat yogurt consumption in the previous year was reported by each participant in the baseline FFQ. Frequency of total yogurt consumption was estimated using the sum of these two food items (whole-fat and low-fat yogurt consumption). The sample was divided into three categories according to their total, whole-fat and low-fat yogurt consumption: 0–250 g/week (0–2 servings/week), >250 to <875 g/week, (>2 to <7 servings/week) and ≥875 g/week (≥7 servings/week); (125 g was considered as one serving size).

### Assessment of non-dietary variables

Other questions (46 items for men and 54 for women) were also included in the baseline questionnaire assessing the participants’ lifestyle, and socio-demographic variables (sex, age, marital status, and employment), health related habits (smoking status, physical activity during leisure time), medical history (prevalence of chronic diseases such as cancer, diabetes and cardiovascular disease), as well as anthropometric data (weight and height) previously validated in the cohort [22]. We calculated body mass index (BMI) as self-reported weight in kilograms divided by the square of the self-reported height in meters. Physical activity was ascertained through a baseline 17-item questionnaire Physical activity was previously validated in a subsample of the cohort, with weekly MET-hours shown to adequately correlate with objectively measured energy expenditure [Spearman ρ = 0.51; 95% confidence interval (CI): 0.232, 0.707] [23].

### Outcome assessment

We defined MetS according to the American Heart Association (AHA) and the International Diabetes Federation (IDF) criteria as outlined in the harmonized definition for MetS [4]. According to this harmonized definition, the MetS diagnosis requires the presence of at least 3 of the 5 criteria: central adiposity (in our study waist circumference: ≥94 cm in males and ≥80 cm in females), hypertriglyceridemia (≥150 mg/dL or condition-specific medication), low levels of high-density lipoprotein cholesterol (<40 mg/dL for men and < 50 mg/dL for women), elevated blood pressure (systolic ≥ 130 and/or diastolic ≥ 85 mmHg or antihypertensive drug treatment in a patient with a history of hypertension), and impaired glucose metabolism (fasting glucose ≥ 100 mg/dL or drug treatment of elevated glucose). For a sensitivity analysis we used a higher cut-off point for central adiposity (waist circumference: ≥102 cm in males and ≥88 cm in females) as proposed by the WHO for Caucasian populations [4].

Self-reported information about each specific MetS criterion was collected in the Q_6 (6th year follow-up questionnaire). With the Q_6 questionnaire, a measuring tape and an explanation of how to measure their own waist were sent to each participant. Diagnosis of MetS itself, the same as each MetS criterion was previously validated in a subsample of the cohort [24,25]. The validation study for MetS diagnosis found agreement between self-reported MetS diagnosis and MetS diagnosis from participant medical records to be 91.2% for confirmed MetS (95% CI: 80.7-97.1%) and 92.2% for non-confirmed MetS (95% CI: 85.1-96.4%) [24].

Incident cases of MetS were defined as all participants who did not have any MetS criterion at baseline, and reported 3 or more criteria of MetS in the 6th year follow-up questionnaire.

### Statistical analyses

Three categories according to their baseline yogurt consumption were used to classify the participants included in the study: 0–250 g/week (0–2 servings/week), >250 to <875 g/week (>2 to <7 servings/week), and ≥875 g/week (≥7 servings/week). The group with the lowest frequency of consumption (0–250 g/week) was considered as the reference category.

Logistic regression models were fitted to assess the relationship between total, whole-fat or low-fat yogurt consumption at baseline and the risk of developing incident MetS during the first 6 years of follow-up. The specific relationship between each of the 5 components of the MetS and total, whole-fat and low-fat yogurt consumption were also assessed. Odds Ratios (OR) and their 95% CI were calculated. Tests for linear trend across increasing categories of yogurt consumption were conducted by assigning the median consumption within each category and treating this variable as a continuous variable.

The interaction between adherence to Mediterranean diet [low (0–4 pts)/high (5–8 pts)], and fruit consumption [low (under the median: <264.5 g/d)/high (above the median: ≥264.5 g/d)] and total yogurt consumption were tested using likelihood ratio tests comparing the fully adjusted model and the same model with the interaction product-term (2 degrees of freedom).

Additional analyses were conducted to test the association of the joint exposure to total yogurt consumption and fruit consumption with the risk of MetS, therefore we built six categories of the joint combined exposure to total yogurt consumption (low, medium and high consumption) and fruit consumption (under the median and at the median or higher) considering as the reference category those who had low yogurt consumption (<250 g/week) and low fruit consumption (under the median).

We fitted a first model without any adjustment (crude), a second model adjusted for age and sex, and a third multivariable-adjusted model after additional adjustment for the following potential confounders: baseline weight (kg), total energy intake (kcal/d), alcohol (g/d), soft drink consumption (ml/d), red meat consumption (g/d), consumption of French fries (g/d), consumption of fast food (g/d), Mediterranean dietary pattern adherence (3 categories: low, medium, and high adherence), physical activity (METs-hours/week), sitting hours (hr/d), sedentary behavior (hr/d), smoking status (non-smoker, smoker, former smoker), snacking between meals (yes/no), and following a special diet (yes/no). Snacking between meals was defined as those participants who responded affirmatively to the question “Do you eat between main meals (snacking)?” We did not consider if it was healthy or unhealthy snacking. The percentage of participants who reported snacking between meals was 33.3%. We considered that a participant followed a special diet, if she/he answered yes to the question “*Do you follow any special diet”.* If they had answered yes, they were asked which kind of special diet they followed (such as hypocaloric diet, vegetarian diet, low-fat diet, low sodium diet, high protein diet, among others). We used this variable dichotomously. The percentage of participants following a special diet was 6.1%.

For the selection of the potential confounders in the multivariate model, and as its currently recommended [26], we took into account the previously published scientific literature including our own results based on the cohort about risk factors for hypertension, overweight/obesity, type 2 diabetes, avoiding exclusively the statistical approach, the stepwise procedures, or the changes in the point estimates after adjusting for potential confounders.

All *p* values presented are two-tailed; *p* < 0.05 was considered statistically significant. Analyses were performed using STATA/SE version 12.0 (StataCorp, College Station, TX, USA).

## Results

The main characteristics of participants according to their frequency of yogurt consumption are presented in Table 1. The mean age of the participants was 36.4 y (SD: 11.6) and the mean BMI was 22.7 kg/m^2^ (SD: 2.7). On average participants in the highest category of total yogurt consumption had the lowest BMI, were more physically active, less likely to be current smokers, spent less hours sitting, and had the highest total energy intake, carbohydrate and protein intake and the lowest fat intake.

**Table 1:** **Baseline characteristics of participants according to their frequency of total yogurt consumption**

	**Consumption of total yogurt grams per week (servings/week)**			
	**0-250 g/wk (0–2 serv/wk)**	**>250 to <875 g/wk (>2 to <7 serv/wk)**	≥**875 g/wk (**≥**7 serv/wk)**	**p value**
**N**	2,689	3,089	2,285	
**Age (y)**	38.4 (11.2)	35.0 (9.7)	35.8 (10.1)	<0.001
**20-40 y (** ***n*** **5429)**	29.1	41.8	29.2	<0.001
**41-60 y (** ***n*** **2492)**	41.3	31.7	27.0	
**≥61 (** ***n*** **142)**	58.5	20.4	21.1	
**Men (%)**	39.1	33.8	29.0	<0.001
**BMI (kg/m** ^**2**^ **)**	22.9 (2.8)	22.7 (2.7)	22.5 (2.7)	<0.001
**Physical activity (METs-h/week)**	19.0 (21.8)	20.6 (21.6)	23.5 (24.5)	<0.001
**Sitting time (h/d)**	5.3 (2.0)	5.4 (2.0)	5.2 (2.1)	0.059
**Tv watching (h/d)**	1.6 (1.2)	1.6 (1.2)	1.6 (1.2)	0.191
**Smoking status (%)**				<0.001
**Current smokers**	27.4	22.9	17.4	
**Former smokers**	28.6	24.9	26.8	
**Total energy intake (kcal/d)**	2,256 (630)	2,379 (591)	2,482 (580)	<0.001
**Macronutrients (% energy)**				
**Carbohydrate intake**	43.1 (7.8)	43.2 (6.8)	44.8 (7.0)	<0.001
**Protein intake**	17.5 (3.5)	17.9 (3.0)	18.3 (3.2)	<0.001
**Fat intake:**	37.2 (6.9)	37.2 (6.1)	35.5 (6.4)	<0.001
**SFA**	12.8 (3.3)	12.9 (2.9)	12.0 (3.1)	<0.001
**MUFA**	16.3 (4.0)	15.9 (3.5)	14.9 (3.5)	<0.001
**PUFA**	5.5 (1.7)	5.3 (1.5)	4.9 (1.5)	<0.001
**Alcohol intake (g/d)**	6.9 (10.0)	5.8 (7.9)	4.9 (7.1)	<0.001
**Soft drinks (ml/d)**	45.0 (93.8)	42.5 (66.3)	38.1 (76.9)	0.008
**Red meat (g/d)**	79.4 (48.6)	81.6 (44.4)	72.9 (44.6)	<0.001
**French fries (g/d)**	28.2 (31.9)	28.0 (29.1)	23.7 (28.1)	<0.001
**Fast food (g/d)**	20.1 (18.8)	22.9 (21.1)	19.4 (18.6)	<0.001
**Adherence to the Mediterranean diet (0[minimum] to 8[maximum] score)**	4.1 (1.7)	4.0 (1.8)	4.1 (1.8)	<0.001
**Between-meals snacking (%)**	31.7	35.1	32.9	0.022
**Following special diets (%)**	6.1	5.4	7.0	0.047

Values are expressed as means (SD), unless otherwise stated. BMI, Body mass index; SFA, saturated fatty acid; MUFA, Monounsaturated fatty acid; PUFA, polyunsaturated fatty acid.

The SUN cohort, 1999–2013.

The association between the frequency of yogurt consumption and the risk of incident MetS was assessed in 8,063 participants initially free of MetS. During the first 6 years of follow up we observed 306 incident cases of MetS. As displayed in Table 2, a higher frequency of total, whole-fat or low-fat yogurt consumption was not significantly associated with a lower risk of developing MetS. Participants consuming total, whole-fat and low-fat ≥875 g/week did not exhibit significantly lower risk of developing MetS compared with those who consumed from 0 to 250 g/week. However, we observed non-significant inverse associations [OR = 0.84 (95% CI: 0.60-1.18); 0.98 (95% CI: 0.68-1.41); and 0.63 (95% CI: 0.39-1.02)] respectively for total, whole-fat and low-fat yogurt consumption, after adjusting for potential confounders (Table 2).

**Table 2:** **Odds Ratio (OR) and 95% confidence intervals (CI) of incident MetS according to baseline frequency of total, whole-fat, and low-fat yogurt consumption in 8,063 participants of the SUN Project (1999–2013)**

	**0-250 g/week (0–2 servings/week)**	**>250 to <875 g/week (>2 to <7 servings/week)**	**≥875 g/week (≥7 servings/week)**	**P for trend**
***Total yogurt***				
**n**	2,689	3,089	2,285	
**Incident cases**	125	117	64	
**Crude**	1.00 Ref.	0.81 (0.62-1.04)	0.59 (0.43-0.80)	0.001
**Age and sex**	1.00 Ref.	1.22 (0.93-1.60)	0.85 (0.61-1.17)	0.315
Multivariable*	1.00 Ref.	1.22 (0.92-1.62)	0.84 (0.60-1.18)	0.319
***Whole fat yogurt***				
**n**	4,160	2,421	1,482	
**Incident cases**	182	81	43	
**Crude**	1.00 Ref.	0.76 (0.58-0.99)	0.65 (0.47-0.92)	0.006
**Age and sex**	1.00 Ref.	1.07 (0.81-1.42)	0.87 (0.61-1.23)	0.544
Multivariable*	1.00 Ref.	1.16 (0.87-1.55)	0.98 (0.68-1.41)	0.885
***Low fat yogurt***				
**n**	5,990	1,242	831	
**Incident cases**	233	52	21	
**Crude**	1.00 Ref.	1.08 (0.79-1.47)	0.64 (0.41-1.01)	0.125
**Age and sex**	1.00 Ref.	1.34 (0.97-1.84)	0.74 (0.47-1.18)	0.647
Multivariable*	1.00 Ref.	1.15 (0.83-1.61)	0.63 (0.39-1.02)	0.169

*Adjusted for: age, sex, baseline weight, total energy intake, alcohol intake, soft drinks, red meat, French fries, fast food, Mediterranean diet, physical activity, sedentary behavior, hours sitting, smoking status, snacking between meals, following special diet.

**Figure 1:**
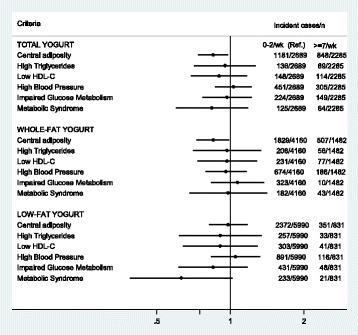
**Multiple adjusted Odds Ratio (** **OR)** * and 95% CI of each MetS criteria for ≥ 875 g/week (≥7 servings/week) of yogurt consumption [compared with ≤250 g/week (≤2 servings/week)]. The SUN Project 1999–2013. *Adjusted for: age, sex, baseline weight, total energy intake, alcohol intake, soft drinks, red meat, French fries, fast food, Mediterranean diet, physical activity, sedentary behavior, hours sitting, smoking status, snacking between meals, following special diet.

The risk of developing each component of MetS according to the total, whole-fat and low-fat yogurt consumption is shown in Figure 1. This figure shows the ORs for each component of the MetS separately for total, whole-fat and low-fat yogurt consumption only for the highest category of consumption (≥875 g/week) versus the lowest category (≤250 g/week). From the 5 components of MetS, we observed that those participants who consumed ≥875 g/week of total and whole-fat yogurt compared with those who consumed ≤ 250 g/week had a lower risk of developing central adiposity [OR = 0.85 (95% CI: 0.74-0.98) and 0.85 (95% CI: 0.73-0.99) respectively, after adjusting for potential confounders. The results for the rest of the components suggested a non-significant trend for a lower risk (except for blood pressure).

As a sensitivity analysis we changed the cut-off point for central adiposity (waist circumference: ≥102 cm in males and ≥88 cm in females) as proposed by the WHO for Caucasian populations. The ORs for total, whole-fat and low-fat yogurt consumption (≥875 g/week versus ≤250 g/week) were 0.74 (95% CI: 0.48-1.14); 0.76 (95% CI: 0.46-1.25); and 0.83 (95% CI: 0.47-1.45), respectively (data not shown).

No interaction between Mediterranean diet adherence (low/high) and total, yogurt consumption was observed (p for interaction = 0.270). The interaction between fruit consumption (below/above median) and total yogurt consumption approached the threshold for statistical significance (p for interaction = 0.070). In any case, the lowest ORs in Table 3 were observed in the groups of participants with both a higher adherence to the Mediterranean diet and higher consumption of total yogurt [OR = 0.77 (95% CI: 0.46-1.30) p for trend = 0.323] and in participants with both above-the-median fruit consumption and high yogurt consumption [OR = 0.69 (95% CI: 0.43-1.10) p for trend = 0.104] (Table 3).

**Table 3:** **Odds Ratio (OR) and 95% confidence intervals (CI) of incident Metabolic Syndrome according to the baseline total yogurt consumption stratified by adherence to the Mediterranean diet and fruits consumption**

	**0-250 g/week (0–2 servings/week)**	**>250 to <875 g/week (>2 to <7 servings/week)**	**≥875 g/week (≥7 servings/week)**	**P for trend**	**P for interaction**
***Stratified by adherence to the Mediterranean diet (MeDiet)***					
					0.270
**Low MeDiet Adherence (0 to 4)**					
**N**	1851	2079	1362		
**Incident cases**	75	78	35		
**Crude**	1.00 Ref.	0.92 (0.67-1.28)	0.62 (0.42-0.94)	0.024	
**Age and sex**	1.00 Ref.	1.43 (1.01-2.02)	0.94 (0.61-1.43)	0.820	
**Multivariable**	1.00 Ref.	1.37 (0.96-1.96)	0.86 (0.55-1.35)	0.538	
**High MeDiet Adherence (5 to 8)**					
**N**	838	1010	923		
**Incident cases**	50	39	29		
**Crude**	1.00 Ref.	0.63 (0.41-0.97)	0.51 (0.32-0.82)	0.006	
**Age and sex**	1.00 Ref.	0.94 (0.60-1.47)	0.75 (0.46-1.22)	0.250	
Multivariable*	1.00 Ref.	0.96 (0.60-1.54)	0.77 (0.46-1.30)	0.323	
***Stratified by fruit consumption***					
					0.070
**Low fruit consumption**					
**N**	1482	1636	914		
**Incident cases**	60	64	30		
**Crude**	1.00 Ref.	0.96 (0.67-1.38)	0.80 (0.51-1.26)	0.343	
**Age and sex**	1.00 Ref.	1.38 (0.94-2.01)	1.12 (0.71-1.78)	0.569	
Multivariable*	1.00 Ref.	1.39 (0.94-2.05)	1.06 (0.65-1.73)	0.750	
**High fruit consumption**					
**N**	1207	1453	1371		
**Incident cases**	65	53	34		
**Crude**	1.00 Ref.	0.67 (0.46-0.96)	0.45 (0.29-0.68)	<0.001	
**Age and sex**	1.00 Ref.	1.08 (0.73-1.61)	0.71 (0.46-1.11)	0.123	
Multivariable*	1.00 Ref.	1.05 (0.70-1.59)	0.69 (0.43-1.10)	0.104	

*Adjusted for: age, sex, baseline weight, total energy intake, alcohol intake, soft drinks, red meat, French fries, fast food, Mediterranean diet (only when stratified by fruit consumption), physical activity, sedentary behavior, hours sitting, smoking status, snacking between meals, following special diet.

When we simultaneously analyzed total yogurt consumption (3 categories) and fruit consumption (2 categories), 6 new categories were created. The category of lower yogurt consumption (≤250 g/week) and low fruit consumption (under the median < 264.5 g/d) was considered as the reference group. We observed a significantly lower risk of developing MetS in the group where a higher yogurt consumption (≥875 g/week) was accompanied by a high fruit consumption (above the median ≥264.5 g/d) OR = 0.61 (95% CI: 0.38-0.99) (Table 4).

**Table 4:** Odds Ratio (OR)* and 95% CI of incident MetS according to baseline frequency of total yogurt consumption and fruit consumption in 8,063 participants of the SUN Project (1999–2013)

**Yogurt consumption**	**Fruit consumption**	
	**Low (<264.5 g/d)**	**High (≥264.5 g/d)**
**Low (≤250 g/wk, ≤2 servings/wk)**		
N/incident cases	60/1482	65/1207
Multivariable	1.00 (Ref.)	0.89 (0.59-1.34)
**Moderate (>250 to < 875 g/wk, >2 to < 7 servings/wk)**		
N	64/1636	53/1453
Multivariable	1.40 (0.95-2.05)	0.92 (0.61-1.42)
**High (≥875 g/wk, ≥7 servings/wk)**		
N	30/914	34/1371
Multivariable	1.07 (0.66-1.72)	0.61 (0.38-0.99)

*Adjusted for: age, sex, baseline weight, total energy intake, alcohol intake, soft drinks, red meat, French fries, fast food, Mediterranean diet, physical activity, sedentary behavior, hours sitting, smoking status, snacking between meals, following special diet.

## Discussion

The present study investigated the association between yogurt consumption (total, whole-fat and low-fat) and the risk of MetS in a relatively young Mediterranean population. The results showed that yogurt consumption itself was not significantly associated with the risk of developing MetS. A higher consumption of total and whole-fat yogurt was found to be associated with a reduced risk of central adiposity after adjusting for potential confounders. Interestingly, we found an inverse association for the combined exposure to both high yogurt consumption and high fruit consumption with the risk of MetS. This association was not confounded by other lifestyle factors or nutritional patterns because it remained statistically significant after multiple adjustments for these potential confounding factors.

Our findings are consistent with some previous cross-sectional studies indicating an inverse but non-significant association between yogurt consumption and MetS [6,10]. Another cross-sectional study, had found a meaningful inverse association [11]. The lack of significance in our study might be explained by the low incidence that we found in our cohort 3.8% in comparison with the 25.8 % of MetS prevalence that other studies have reported [11]. Furthermore, the findings of cross-sectional studies should be interpreted with caution because this design, by nature, is more prone to reverse causality bias.

There are some reports in the literature relating the consumption of dairy --and in some cases yogurt consumption-- with some specific isolated criteria of the MetS (central adiposity, low high-density lipoprotein cholesterol, elevated blood pressure, and impaired glucose metabolism or type 2 diabetes). In this context, when we analyzed separately each component of the MetS, we found a significant inverse association between total and whole-fat yogurt consumption and the incidence of central adiposity. This association might be explained by the theory that calcium intake can reduce lipogenesis and increase lipolysis. Therefore, an increased intake of calcium contained in yogurt might lead to an increase in fat oxidation [27,28]. A relationship between microbiota and obesity has been reported by some studies. It is thought that this effect could be mediated by actions such as inflammatory/anti-inflammatory effects of microbiota. *Lactobacillus* and *Bifidobacterium* that come from dairy products, including yogurt, are suggested to positively modulate the gut microbiota and may help to prevent or treat some diseases [29].

Our longitudinal results are novel, but they are in some ways consistent with 2 previous long-term prospective studies that did not specifically assess MetS. The first study was conducted by Mozaffarian et al. [30] using data from three large American cohorts. They found that yogurt consumption was inversely associated with weight gain during the follow-up [−0.82 lb/serving (95% CI: −0.99 to −0.67 lb/serving) in a 4-y period]. Additionally, Wang et al. [31], as a part of the in the Framingham Heart Study Offspring, found that yogurt consumption was also associated with less average weight gain (p for trend = 0.03).

The second component of MetS that was analyzed was impaired glucose metabolism or type 2 diabetes. We did not find any significant association between yogurt consumption at baseline with the likelihood of meeting this criterion after 6 of follow-up. One recent meta-analysis [32] found that consumption of 50 g/d of yogurt was associated with a lower risk of developing this component, finding a pooled RR of 0.91 (95% CI: 0.82–1.00). The average age of participants in the studies included in that meta-analysis was in general over 50 years, whereas the mean age of participants in our cohort was 36.4 years. This difference may explain the apparent null association between yogurt consumption and glucose abnormalities in our cohort, and the discrepancies between our study and the meta-analysis.

For the two lipid components of the metabolic syndrome (high triglycerides and low HDL-C) we did not find any significant association with frequent yogurt consumption, even though, in all cases our non-significant estimates were in the direction of an inverse association. Our results are in the same direction as those found by Kim et al. [10], who reported an inverse but not significant association between yogurt consumption and these 2 components of the MetS.

Finally, when we analyzed high blood pressure, we did not find any association with frequent yogurt consumption. These results might seem contradictory to the results previously found in our SUN cohort by Alonso et al. [33]. In that study we reported that a low-fat “dairy” consumption, but not whole-fat dairy consumption, was associated with a lower risk of incident hypertension. In that study, skim and partially skim milk were the major contributors to low-fat dairy consumption and accounted for 92% of the total low-fat dairy consumption. By contrast, in the current assessment we only evaluated yogurt consumption and not milk. However, we conducted a secondary analysis including all low-fat dairy products (including partially skim milk, skim milk, skim yogurt, cottage cheese and white fresh cheese) and observed an inverse and significant association between low-fat dairy consumption and the incidence of hypertension [OR = 0.81 (95% CI: 0.67-0.98)] for the highest quintile of consumption compared with the lowest, after adjusting for potential confounders (data not shown).

When we analyzed the association between total yogurt consumption and the risk of MetS, stratifying participants according to the adherence to the Mediterranean dietary pattern or according to fruit consumption there was a suggestion of a lower risk of developing MetS among participants with a higher adherence to the Mediterranean dietary pattern, and also among those with high fruit consumption. These results are in accordance with the results reported by Joung et al. [15], who found that a “fruit, salad, cereal and fish” pattern were negatively but not statistically associated with the risk of MetS.

When we simultaneously analyzed total yogurt consumption and fruit consumption, we found that the category of a combined exposure to both higher yogurt consumption and high fruit consumption was significantly associated with a lower risk of developing MetS. This is in agreement with the conclusion of a review study conducted by Baxter et al. [13] that suggested that it is more likely that the interactions between many components of the diet or the overall quality of a dietary pattern offer protection against MetS than each of the isolated food components.

The self-reported outcome might be considered as a major limitation of our study. However, the MetS diagnosis itself [24] and each one of the 5 self-reported MetS components [25] were previously validated in our cohort, finding intraclass correlation coefficients between 0.5 to 0.9 (p < 0.001) depending on the criteria, against a gold standard of direct assessments by an experienced physician. Self-reported weight and BMI were also validated in a subsample of the cohort, finding a mean relative error for weight and BMI of 1.45% and 2.64% respectively [22]. Although we have adjusted for potential confounding factors, since this is an observational study, we cannot completely rule out the possible existence of residual confounding.

The present study has several strengths, including the prospective design, which limits the possibility of reverse causality bias, the use of previously validated methods published in reputed journals [17-19,22-25], the large sample size, and the high educational level of our participants which allows a better understanding of the questionnaire and reduces the potential for misclassification bias. Although, we should be cautious with respect to the generalization of the results.

## Conclusions

The findings of this study showed that high consumption of yogurt at the same time with a high consumption of fruits was inversely associated with the development of MetS. Additionally, no association between yogurt consumption with MetS was observed, and only one component out of 5, central adiposity, was inversely associated with high yogurt consumption in this Mediterranean population.

## Abbreviations

SUN: Seguimiento Universidad de Navarra
MetS: Metabolic Syndrome
OR: Odds Ratio
HDL-C: High-density lipoprotein cholesterol
FFQ: Food frequency questionnaire
BMI: Body mass index
MET: Metabolic equivalent index
AHA: American Heart Association
IDF: International Diabetes Federation
Q_6_: 6th year follow-up questionnaire
